# Poster Session II A323 FECAL MICROBIOTA TRANSPLANTATION FOR SYMPTOM IMPROVEMENT IN PATIENTS WITH IRRITABLE BOWEL SYNDROME: SYSTEMATIC REVIEW AND META-ANALYSIS OF RANDOMIZED CONTROLLED TRIALS

**DOI:** 10.1093/jcag/gwaf042.323

**Published:** 2026-02-13

**Authors:** N Aumpan, J Watanabe, Y Yuan, T Kanno, G Leontiadis, F Chan, P Moayyedi

**Affiliations:** Farncombe Family Digestive Health Research Institute, McMaster University, Hamilton, ON, Canada; Farncombe Family Digestive Health Research Institute, McMaster University, Hamilton, ON, Canada; Farncombe Family Digestive Health Research Institute, McMaster University, Hamilton, ON, Canada; Farncombe Family Digestive Health Research Institute, McMaster University, Hamilton, ON, Canada; Farncombe Family Digestive Health Research Institute, McMaster University, Hamilton, ON, Canada; Faculty of Medicine, The Chinese University of Hong Kong, NT, Hong Kong; Farncombe Family Digestive Health Research Institute, McMaster University, Hamilton, ON, Canada

## Abstract

**Background:**

Fecal microbiota transplantation (FMT) could improve symptoms of irritable bowel syndrome (IBS) in some previous trials. We updated a prior meta-analysis of randomized controlled trials (RCTs) determining this issue.

**Aims:**

To assess the efficacy of FMT versus placebo for symptom improvement in IBS patients.

**Methods:**

We searched MEDLINE, Embase, and Cochrane CENTRAL from inception to June 25^th^ 2025 for potential studies. We also searched abstracts from the conference proceedings from inception to June 25^th^ 2025. We included RCTs that reported the proportion of patients with IBS symptom improvement assessed between 4 and 24 weeks after FMT. The control arm could receive either autologous FMT or inert placebo. The primary outcome was the proportion of patients with improved IBS symptoms. Secondary outcomes were differences between groups in IBS scores, quality of life scores, and adverse events. Risk ratios (RRs) and standardized mean differences (SMDs) with 95% confidence intervals (CIs) were calculated for dichotomous and continuous outcomes, respectively. Risk of bias was assessed by the Cochrane Risk of Bias tool version 2. Data were pooled using a random effects model.

**Results:**

Twelve RCTs with 663 patients were eligible and we have contacted the authors and are awaiting data from one paper. FMT demonstrated no significant improvement in IBS symptoms at 12 weeks post-treatment compared to placebo (RR symptoms not improving with FMT = 0.71; 95% CI 0.48–1.03) (Figure 1). There were no differences in IBS score (SMD -0.4, 95% CI -0.84–0.04) and quality of life score (SMD 0.17, 95% CI -0.27–0.62) between the FMT and placebo group. Overall adverse events were not different in both groups (RR 0.98; 95% CI 0.74–1.29). One serious adverse event (transient vertigo and nausea) occurred in the FMT group and two (acute cholecystitis, suicide) in the placebo group (RR 0.43; 95% CI 0.07–2.69). The subgroup analysis reported significant symptom improvement in the group using FMT via colonoscopy (RR 0.73; 95% CI 0.54–0.99), single transplantation (RR 0.64; 95% CI 0.42–0.99), FMT from single donor (RR 0.56; 95% CI 0.34–0.94), and when IBS was diagnosed by Rome IV criteria (RR 0.24; 95% CI 0.16–0.35). There were no differences in symptom improvement between groups based on type of control group, IBS subtype, risk of bias of included studies, treatment duration, and timing of outcome assessment.

**Conclusions:**

This systematic review suggested overall, there was no significant difference between FMT and placebo for improvement of IBS symptoms. FMT was effective in some subgroup analyses but this needs to be interpreted with caution and more data are needed.

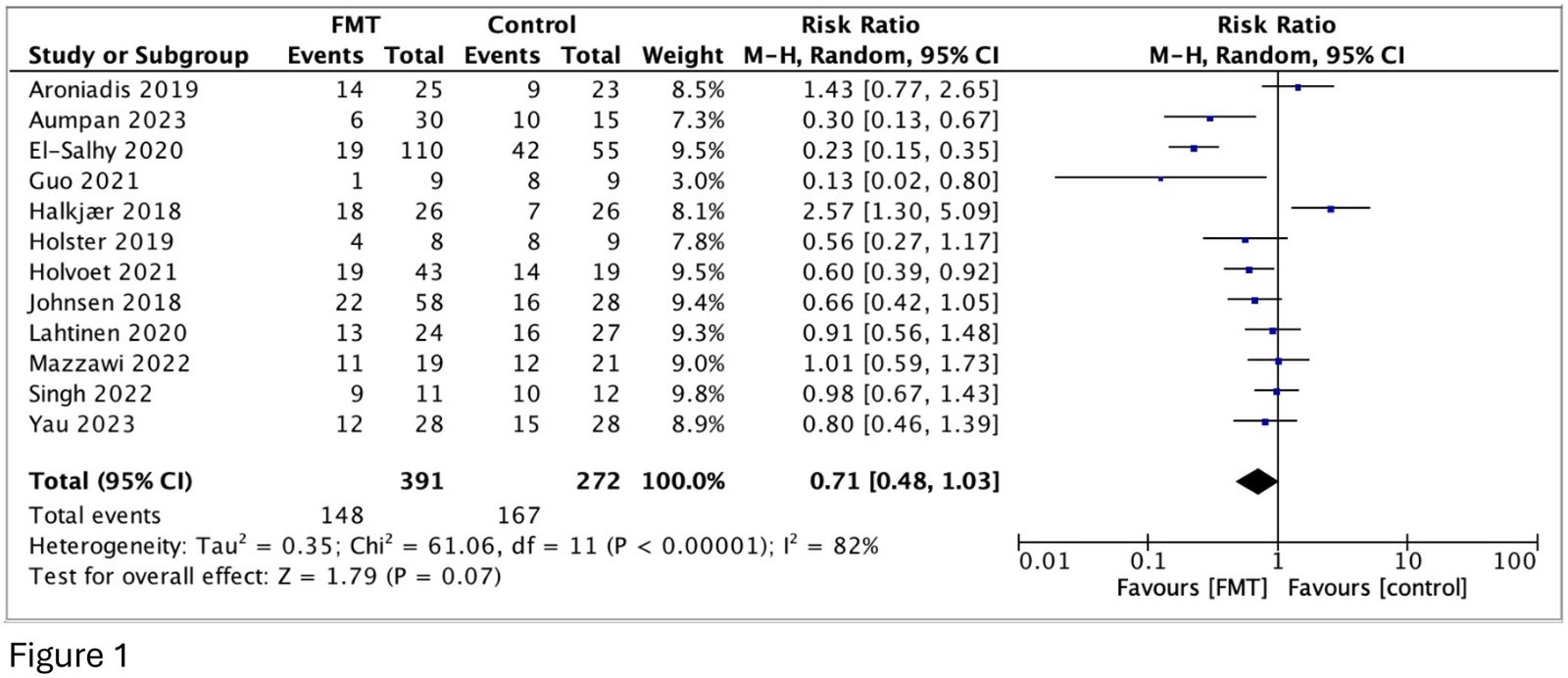

**Funding Agencies:**

None

